# Decreased sarcoplasmic reticulum phospholipids in human skeletal muscle are associated with metabolic syndrome

**DOI:** 10.1016/j.jlr.2024.100519

**Published:** 2024-02-13

**Authors:** Samantha E. Adamson, Sangeeta Adak, Max C. Petersen, Dustin Higgins, Larry D. Spears, Rong Mei Zhang, Andrea Cedeno, Alexis McKee, Aswathi Kumar, Sudhir Singh, Fong-Fu Hsu, Janet B. McGill, Clay F. Semenkovich

**Affiliations:** 1 Division of Endocrinology, Metabolism & Lipid Research, Washington University, St Louis, MO, USA; 2 Department of Cell Biology & Physiology, Washington University, St Louis, MO, USA

**Keywords:** phospholipids/phosphatidylcholine, diabetes, muscle, insulin resistance

## Abstract

Metabolic syndrome affects more than one in three adults and is associated with increased risk of diabetes, cardiovascular disease, and all-cause mortality. Muscle insulin resistance is a major contributor to the development of the metabolic syndrome. Studies in mice have linked skeletal muscle sarcoplasmic reticulum (SR) phospholipid composition to sarcoplasmic/endoplasmic reticulum Ca^2+^-ATPase activity and insulin sensitivity. To determine if the presence of metabolic syndrome alters specific phosphatidylcholine (PC) and phosphatidylethanolamine (PE) species in human SR, we compared SR phospholipid composition in skeletal muscle from sedentary subjects with metabolic syndrome and sedentary control subjects without metabolic syndrome. Both total PC and total PE were significantly decreased in skeletal muscle SR of sedentary metabolic syndrome patients compared with sedentary controls, particularly in female participants, but there was no difference in the PC:PE ratio between groups. Total SR PC levels, but not total SR PE levels or PC:PE ratio, were significantly negatively correlated with BMI, waist circumference, total fat, visceral adipose tissue, triglycerides, fasting insulin, and homeostatic model assessment for insulin resistance. These findings are consistent with the existence of a relationship between skeletal muscle SR PC content and insulin resistance in humans.

Metabolic syndrome affects more than 40% of adults and is associated with greater all-cause mortality as well as substantial medical care costs (1, 2, 3). Understanding the pathogenesis of metabolic syndrome may provide insight into improving human health. Skeletal muscle is responsible for a large proportion of insulin-stimulated glucose utilization, and muscle insulin resistance is a chief contributor to the development of metabolic syndrome (4). Abnormalities in lipid metabolism, including increased fatty acid delivery to skeletal muscle, accumulation of toxic bioactive lipids such as diacylglycerol and ceramides, and ectopic intramyocellular triglyceride storage, have been linked to insulin resistance (5, 6, 7). Phospholipids, which have vital biophysical, cellular, and metabolic functions, may also contribute to the pathogenesis of muscle insulin resistance (8, 9).

Phosphatidylcholine (PC) and phosphatidylethanolamine (PE), predominant lipid species in cell membranes, have asymmetric distributions with PC found mostly in the outer leaflet and PE found mostly in the inner leaflet. Alterations in PC and PE, specifically the ratio of PC to PE, can impact membrane integrity and function of membrane-localized enzymes such as SERCA (sarcoendoplasmic reticulum calcium ATPase), which is responsible for sequestering calcium from the cytosol into the sarcoplasmic reticulum (SR). In mice, muscle-specific deletion of *Cept1*, the enzyme responsible for the final step in PC and PE synthesis, decreases SR PE but not PC concentrations, resulting in an increased PC:PE ratio, impaired SERCA-mediated SR calcium uptake, and improved insulin sensitivity because of activation of AMP-activated protein kinase (10). In a mouse model of reduced SERCA activity because of muscle overload, increased PC but not PE levels were observed, resulting in an increased PC to PE ratio that did not reach statistical significance (11).

In humans, assessments of the relationship between skeletal muscle phospholipids and insulin resistance have produced mixed results. Both skeletal muscle PC and PE content were positively correlated with insulin sensitivity, and the PC:PE ratio was negatively correlated with insulin sensitivity, in a cohort study across a broad range of metabolic health, including endurance-trained athletes, sedentary people with obesity, and sedentary people with diabetes (12). In this study, the PC:PE ratio was significantly higher in muscle from participants with diabetes compared with obese and endurance-trained athlete groups. Similarly, in a study of healthy, normal weight people, PC (but not PE) was positively correlated with insulin sensitivity (13). However, in a study of dysglycemic overweight subjects compared with normal weight controls, there were no differences in resting skeletal muscle PC, PE, or PC:PE ratio (14). Exercise training in these overweight men increased skeletal muscle PC and PE levels and decreased PC:PE ratio (14), and the same effects of exercise were reported in obese South African women (15). A single bout of exercise decreased skeletal muscle PC and PE in endurance-trained athletes but not obese or diabetic groups, but PC:PE remained unchanged (12). In human muscle samples, *CEPT1* expression was negatively correlated with insulin sensitivity in a group of participants of varying metabolic health (10). Consistent with this finding, human fatty acid synthase and CEPT1 protein contents in muscle were decreased in patients who had significant weight loss after bariatric surgery (16).

Previous studies in humans have measured phospholipids in whole-muscle lysate rather than specific subcellular compartments. Building on previous work linking SR phospholipids to SERCA activity and muscle insulin action, we hypothesized that SR PC and PE concentrations would be altered and the PC:PE ratio would be decreased in people with metabolic syndrome. To test this hypothesis, we compared SR composition in skeletal muscle from sedentary patients with metabolic syndrome (in the absence of diabetes) and sedentary lean controls without metabolic syndrome.

## Materials and methods

### Study participants and design

The data reported here were obtained from 15 sedentary nonobese people and 12 sedentary people with metabolic syndrome. Data are presented for two cohorts because sample collection and MS analyses were conducted at two points in time separated by several years because of the interruption of the study by a pandemic. Cohort 1 was the larger cohort studied more recently, whereas cohort 2 included a smaller number of participants with samples analyzed before the pandemic. Inclusion criteria for both groups included age 18–65 and sedentary lifestyle, defined as less than 15 min of exercise less than 2 days per week. Inclusion criteria for the sedentary nonobese group included BMI less than 30 kg/m^2^, normal physical exam, and normal fasting glucose, comprehensive metabolic panel, complete blood count, and lipid panel.

Inclusion criteria for the metabolic syndrome group included BMI greater than 30 kg/m^2^ and meeting at least three of the following criteria for metabolic syndrome: *1*) waist circumference greater than 40 inches in men, greater than 35 inches in women; *2*) blood triglycerides greater than 150 mg/dl; *3*) blood HDL cholesterol less than 40 mg/dl in men, less than 50 in women; *4*) blood pressure greater than 130 mm Hg systolic or greater than 85 mm Hg diastolic; and *5*) fasting plasma glucose greater than 100 mg/dl. No participants were pregnant or had evidence of chronic illness, significant organ dysfunction, type 2 diabetes, cardiovascular disease, cancer, or chronic kidney disease. None took medications that interfere with insulin action or glucose metabolism, and none consumed tobacco products and/or excessive amounts of alcohol. Participants in the sedentary nonobese group using medications other than oral contraceptives or antidepressants were excluded. Participants in the metabolic syndrome group using medications other than oral contraceptives, antidepressants, or greater than two standard antihypertensive medications were excluded. Written informed consent was obtained from all participants before their participation. This work abides by the Declaration of Helsinki principles. The experimental protocol (ClinicalTrials.gov no.: NCT02122666) was approved by the Human Research Protection Office at Washington University School of Medicine in St Louis.

All participants underwent a screening visit that included a comprehensive medical evaluation, with a history and physical examination, standard blood tests including fasting glucose, hemoglobin A1c, lipid panel, comprehensive metabolic panel, and complete blood count. Participants had two follow-up phone visits to verify weight stability and that there were no changes in eating habits, exercise, or medications. At the final visit in the Clinical Translational Research Unit, participants completed a dual-energy X-ray absorptiometry (DEXA; Lunar iDXA, GE Healthcare Lunar, Madison, WI) scan to determine body composition, blood tests, and an oral glucose tolerance test with insulin levels. A tissue sample of approximately 100 mg was obtained from the vastus lateralis using a Rongeur after local instillation of 2% lidocaine, immediately immersed in liquid nitrogen, and stored at −80°C.

### Tissue fractionation, phospholipid extraction, and MS

SR was partially purified using a modification of a differential centrifugation protocol known to yield a SERCA activity-enriched fraction in mammals (16, 17). Skeletal muscle samples were homogenized at 4°C using a motor-Teflon pestle in hypotonic buffer (250 mM sucrose, 10 mM NaHCO_3_, 5 mM NaN_3_, 0.1 mM PMSF, and 1× Halt™ protease and phosphatase inhibitor cocktail [Thermo Fisher]), then rotated at 4°C for 1 h, followed by abbreviated differential centrifugations: 1,300 *g* for 10 min for separating the nuclear fraction, 10,000 *g* for 10 min for separating the mitochondrial fraction, and 225,000 *g* for 2 h to isolate the SR and leaving the supernatant with mostly cytosolic contents. Validation of the SR preparation was conducted using skeletal muscles biopsies from two control subjects recruited for a different study and stored frozen at −80^o^C for longer than any of the samples processed for phospholipids. Starting lysate and the supernatant and SR pellet from the final centrifugation step were subjected to Western blotting by standard techniques using a SERCA2 antibody (Abcam; catalog no.: ab3625) and a p70 S6 kinase antibody (Cell Signaling; catalog no.: 9202). Corresponding secondary antibodies were incubated for 1 h at room temperature followed by imaging with a Licor Odyssey FC System and quantitation using Licor Image Studio software (version 5.2).

Samples reconstituted in ddH_2_O were mixed with extraction buffer (2:2 [v/v] chloroform/methanol) in the presence of the internal standards 14:0/14:0-PC (*m/z* 684.5 [M + Li]^+^) or 14:0/14:0-PE (*m/z* 634.5 [M − H]^−^). The organic phase was collected, concentrated to dryness under nitrogen, and reconstituted in methanol. An aliquot was removed, diluted with methanol containing 0.6% LiCl, and analyzed by direct injection electrospray ionization MS on a Thermo Vantage triple-quadruple mass spectrometer in positive mode for the analysis of PC (neutral loss of 183) species. Another aliquot was analyzed in negative mode for PE species. Intensities of individual species were compared with internal standards, and results were generated using a standard curve.

To assign phospholipid structure, high-resolution (*R* = 100,000 at *m/z* 400) and low-energy collision-induced dissociation (CID) LIT MS^n^ analyses were conducted on a Thermo Scientific (San Jose, CA) LTQ Orbitrap Velos mass spectrometer (MS) with Xcalibur operating system. Lipid extracts were dissolved in 0.5% NH_4_OH in methanol and loop injected onto the ESI source and analyzed in the negative-ion mode. The skimmer of the source was set at ground potential, the electrospray needle was set at 4.0 kV, and temperature of the heated capillary was 300°C. The automatic gain control of the ion trap was set to 5 × 10^4^, with a maximum injection time of 50 ms. Helium was used as the buffer and collision gas at a pressure of 1 × 10^−3^ mbar (0.75 mTorr). The MS^n^ experiments were carried out with an optimized relative collision energy ranging from 25–35%, an activation *q* value of 0.25, and an activation time of 10 ms that leave minimal residual precursor ions with abundance around 20%. The mass selection window for the precursor ions was set at 1 Da wide to admit the monoisotopic ion to the ion trap for CID for unit resolution detection in the ion-trap or high-resolution accurate mass detection in the Orbitrap mass analyzer. Mass spectra were accumulated in the profile mode, typically for 2–10 min for MS^n^ spectra (*n* = 2, 3). Structures for PC, PE, and phosphatidylserine (PS) were assigned using a home-built database derived from analysis of eukaryotic lipids. The structures for major PC and PE species were confirmed by CID as described in the Results section.

### Statistical analyses and data inclusion

Statistical analyses were performed by using Prism 8 (GraphPad Software, San Diego, CA). Intra-abdominal adipose tissue was measured in 11 controls and 10 metabolic syndrome subjects instead of the full group because intra-abdominal adipose tissue measurement was not available on the earlier DEXA scan analysis. For insulin levels during oral glucose tolerance test, homeostatic model assessment for insulin resistance (HOMA-IR) and Matsuda index, 11 metabolic syndrome subjects are reported because of missing serum insulin samples for one participant. For phospholipid analysis of cohort 1 in Fig. 5B, C, one metabolic syndrome sample was excluded because phospholipid levels were greater than 1.9 standard deviations higher than the remainder of the samples. For phospholipid data from cohort 2 with control *n* = 3 and metabolic syndrome *n* = 4 in Fig. 5D, E, two control samples and one metabolic syndrome sample are not included because they were lost during tissue processing.

## Results

**Fig. 1:**
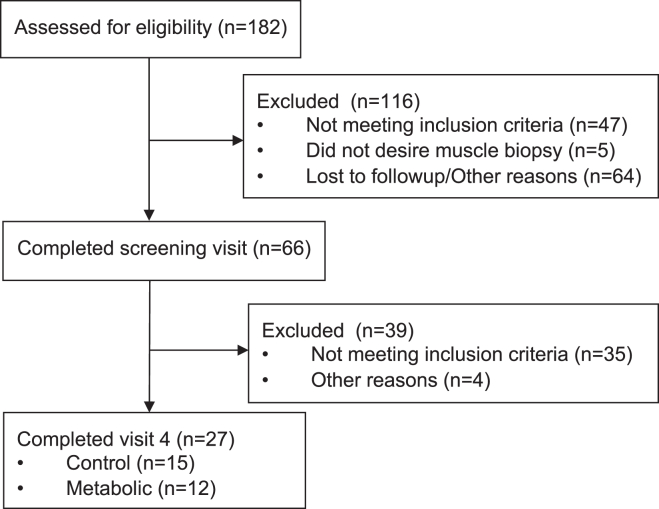
CONSORT diagram.

**Fig. 2:**
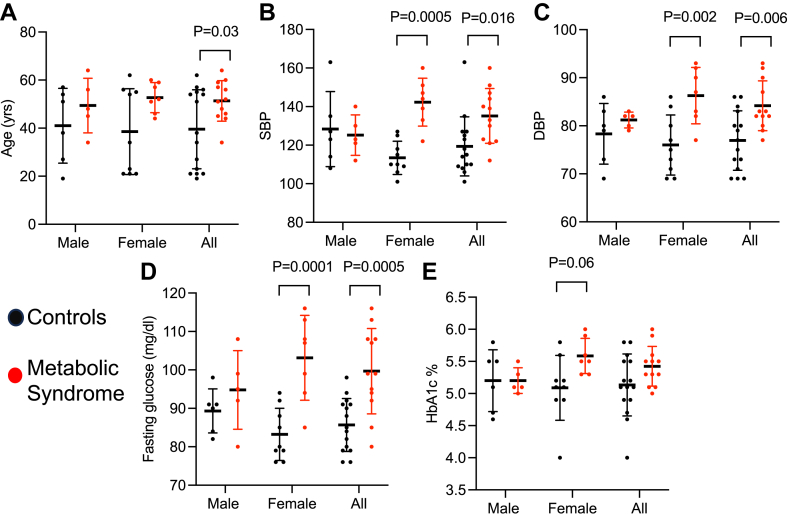
Age (A), systolic blood pressure (SBP) (B), diastolic blood pressure (DBP) (C), fasting glucose (D), and hemoglobin A1c (E) of male, female, and all participants in control and metabolic syndrome groups. Each symbol represents a participant. Error bars indicate standard deviation. Analyzed by two-way ANOVA with Sidak’s multiple comparison test.

Of 182 subjects assessed for eligibility, 27 participants completed the experimental protocol (Fig. 1). Baseline characteristics of participants are presented in supplemental Table S1. The control group was younger than the metabolic syndrome group. Figure 2A shows the distribution of ages of participants in the group. Several younger female participants in the control group resulted in the significantly younger age of the control group. Each group was roughly 40% male and predominantly white. BMI, waist circumference, systolic and diastolic blood pressure, fasting glucose, and triglycerides were all significantly elevated in the metabolic syndrome group, but hemoglobin A1c, total cholesterol, LDL, and HDL were not different between groups (supplemental Table S1). For blood pressure and fasting glucose, the significant increases in the metabolic syndrome group were driven by differences in female participants (Fig. 2B–E).

**Fig. 3:**
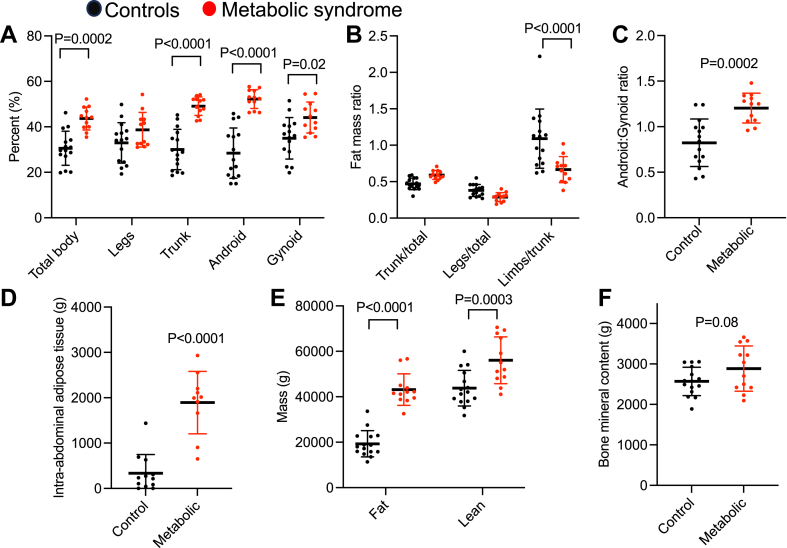
Fat mass assessed by DEXA is significantly increased in metabolic syndrome group compared with control group. A: Percent fat of total body, legs, trunk, android, and gynoid regions. B: Fat mass ratio of trunk to total, legs to total, and limbs to trunk. For (A, B, and E), two-way ANOVA with Sidak’s multiple comparison test. C: Android to gynoid fat mass ratio. Unpaired Student’s *t*-test. D: Intra-abdominal adipose tissue. Control *n* = 11, metabolic *n* = 10. Mann-Whitney test. E: Absolute fat and lean mass. F: Bone mineral content. Unpaired Student’s *t*-test. Each symbol represents a participant. Error bars indicate standard deviation.

DEXA analysis revealed significantly increased percent fat in the total body, trunk, android, and gynoid regions of participants in the metabolic syndrome group compared with control (Fig. 3A). The limbs to trunk fat mass ratio was significantly decreased, whereas the android to gynoid fat mass ratio was significantly increased in the metabolic group (Fig. 3B, C). Intra-abdominal adipose tissue content was significantly higher in the metabolic syndrome group (Fig. 3D). Absolute lean mass was significantly higher in the metabolic syndrome group, but there was no difference in bone mineral content (Fig. 3E, F).

**Fig. 4:**
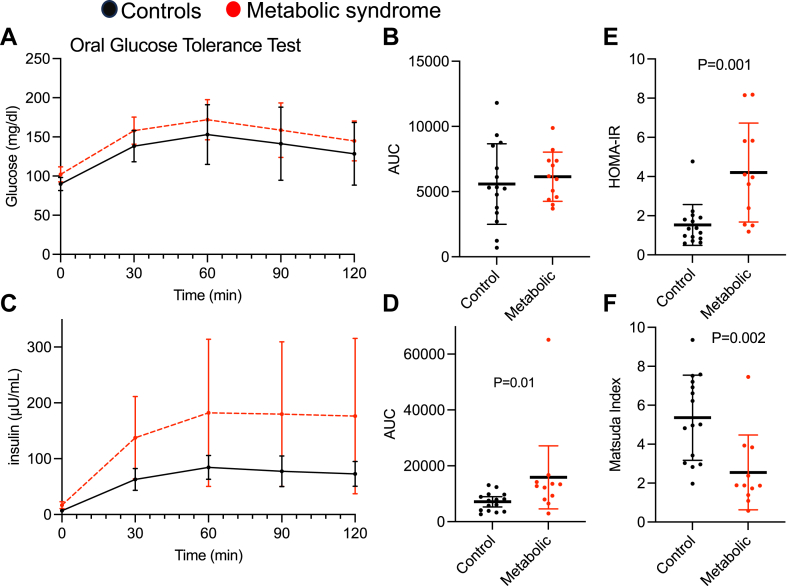
A: Oral glucose tolerance test, data shown as mean, error bars indicate standard deviation. B: Area under the curve (AUC) of oral glucose tolerance test, each symbol represents a participant. Error bars indicate standard deviation. C: Insulin levels during oral glucose tolerance test. D: AUC of insulin levels, data shown as mean, error bars indicate 95% confidence interval. Mann-Whitney test. Each symbol represents a participant. E: HOMA-IR in participants. Unpaired Student’s *t*-test with Welch’s correction, each symbol represents a participant. F: Matsuda index in participants. Unpaired Student’s *t*-test. Each symbol represents a participant.

There was no significant difference in oral glucose tolerance tests between control and metabolic syndrome groups (Fig. 4A, B). However, insulin levels during the oral glucose tolerance test were significantly higher in the metabolic syndrome group (Fig. 4C, D). The metabolic syndrome group was significantly more insulin resistant than the control group by HOMA-IR (Fig. 4E) and Matsuda index (Fig. 4F).

**Fig. 5:**
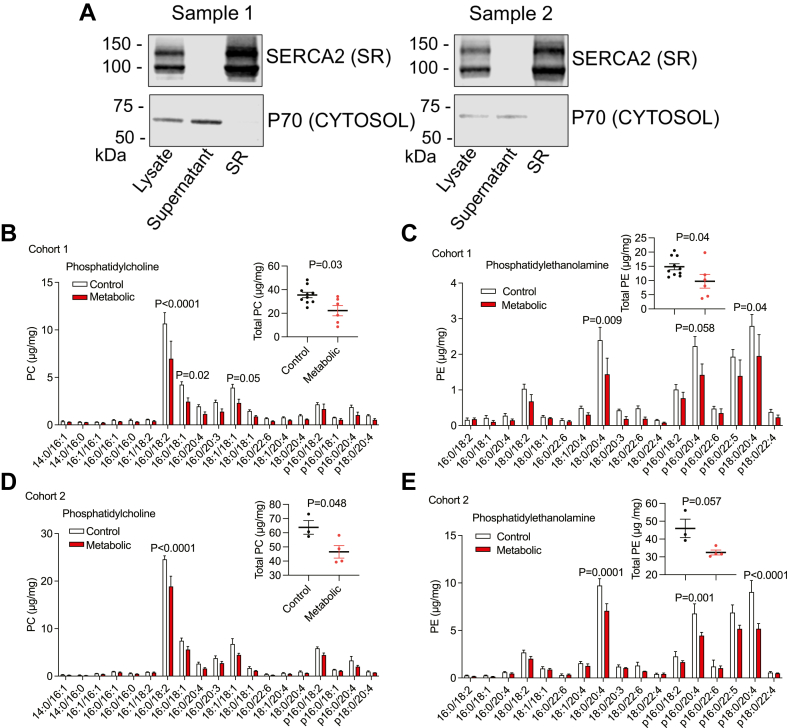
A: Demonstration of partial purification of SR from skeletal muscle samples by Western blotting. Samples 1 and 2 were obtained from two different control subjects recruited for a different study and stored frozen for longer than cohort 1 and cohort 2 samples processed for phospholipids. Blots were detected with the SR marker SERCA2 and the cytosolic marker p70 S6 kinase. B, D: PC levels in SR of muscle from cohort 1 and cohort 2. C, E: PE levels in SR of muscle from cohort 1 and cohort 2. Data shown as mean, error bars indicate standard error of the mean. Individual phospholipid species analyzed by two-way ANOVA with Bonferroni multiple comparison test. Insets show total PC and PE levels, with data shown as mean, error bars represent standard error of the mean, analyzed by Mann-Whitney test, each symbol represents a participant sample. Cohort 1: control *n* = 10, metabolic syndrome *n* = 6. Cohort 2: control *n* = 3, metabolic syndrome *n* = 4.

Skeletal muscle samples obtained from the vastus lateralis muscle were processed to isolate SR-enriched fractions from which lipids were extracted for analysis by MS. To confirm the isolation of SR from stored samples, muscle biopsies from two independent control subjects (participating in a different study) that were stored at −80^o^ for longer than any of the samples from subjects in the current study were processed for SR, and immunoblot markers of SR purity were assayed. As shown in Fig. 5A, the SR marker SERCA2 was not detected in supernatants (enriched in cytosolic contents) from the final centrifugation for sample 1 and sample 2. SERCA2 intensity in the SR fraction increased by 65% as compared with the initial lysate for sample 1 and increased by 63% as compared with the initial lysate for sample 2. The cytosolic marker p70 S6 kinase was not detected in either of the SR fractions.

We applied ESI high-resolution MS on the PC lipid family from SR samples, detected as [M + H]^+^ ions in the positive ion mode (supplemental Table S2). Structures are assigned using a home-built database library established by CID MS^n^ on eukaryotic lipids. LIT MS^2^ on PC [M + H]^+^ ions yielded a predominated ion at *m/z* 184, representing a phosphocholine ion unique to the PC species, but structural information regarding the fatty acyl chains and their regiospecificity (sn-1 and sn-2) are absent (18). To confirm the structural assignments, we applied MS^3^ on the corresponding [MX – CH_3_X]^−^ (X = CH_3_CO_2_, HCO_2_, Cl) (in the present case, X = HCO_2_^−^ because of the presence of NH_4_OCO_2_) adduct ion in the negative ion mode for structural assignment (19, 20) using control samples. For example, CID MS^3^ on ions of *m/z* 742 (802 → 742; supplemental Fig. S1A) and 770 (830 → 770; supplemental Fig. S1B) yielded fragment ions readily pointing to 16:0/18:2-PC and 18:0/18:2-PC structures, respectively. These results are in accord with the structural assignment extracted from our home-built database search that demonstrates the [M + H]^+^ ions at *m/z* 758 and 786 represent 16:0/18:2-PC and 18:0/18:2-PC, respectively (supplemental Table S2). To verify the 1-O-alkyl-, 1-O-alkenyl-, and 1-O-acyl chain at sn-1 of the PC molecules, we applied MS^n^ (*n* = 2, 3, 4) on the [M + HCO_2_]^−^ adduct ions. CID MS^2^ on the [M + HCO_2_]^−^ ions at *m/z* 786 (corresponding to the [M + H]^+^ at *m/z* 742) gave rise to *m/z* 726 (supplemental Fig. S2A) by loss of HCO_2_CH_3_ (60 Da). MS^3^ on ions of 726 (786 → 726; supplemental Fig. S2B) yielded *m/z* 464, which further dissociated to the feature ions (786 → 726 → 464; supplemental Fig. S2C) that define a p16:0/18:2-PC structure (supplemental Fig. S2D) (20). We also obtained the MS^n^ spectra on other major PC species (supplemental Figs. S3 and S4) to confirm assigned structures. The diacyl-PC structures are consistent with those extracted from our home-built database. However, plasmalogen PCs are the major ether PC lipids, which differs from murine tissues, in which plasmanyl-PCs are abundant (20).

We applied ESI high-resolution MS on the PE lipid family from SR samples, detected as [M − H]^−^ ions in the negative ion mode (supplemental Table S3). To confirm structural assignments, we applied CID MS^n^ on the major PE species in control samples at *m/z* 766 and 742 (supplemental Fig. S5), 750 (supplemental Fig. S6), 748 (supplemental Fig. S7), 722 (supplemental Fig. S8), and 698 and 726 (supplemental Fig. S9) (19, 21). These structures are consistent with those extracted from our home-built database.

Data are presented for two cohorts because sample collection and MS analyses were conducted at two points in time separated by several years because of the interruption of the study by a pandemic. For the larger cohort studied more recently (cohort 1), total PC levels were significantly decreased in the SR-enriched fractions of muscle samples from the metabolic syndrome group compared with controls (Fig. 5B). The two most abundant PC species, 16:0/18:2 and 16:0/18:1, were significantly decreased in the metabolic group. Similarly, total PE levels were significantly decreased in the SR-enriched fractions of muscle samples from the metabolic syndrome group compared with controls (Fig. 5C). There was no significant difference in PS levels between groups in cohort 1 (supplemental Fig. S10A), but PS levels were significantly decreased in the metabolic group in the smaller cohort 2 (supplemental Fig. S10B). Analyzing the phospholipid data based on sex for cohort 1 showed significantly decreased PC and PE in female metabolic syndrome participants compared with female controls (supplemental Fig. S11A, B), but there were no differences in PC, PE, or PS between male metabolic syndrome participants and male controls (supplemental Fig. S11D–F).

**Fig. 6:**
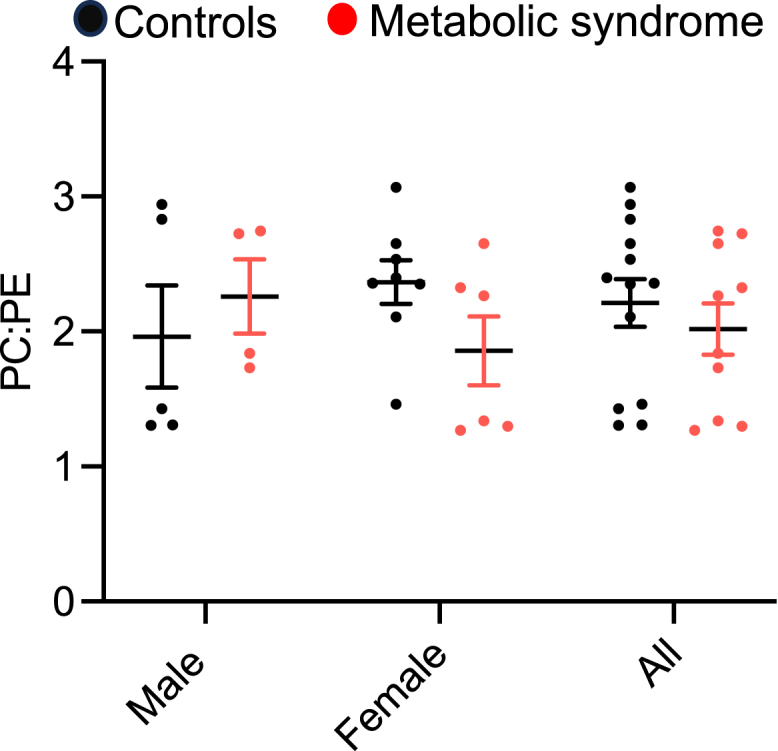
PC to PE ratio in SR of muscle from control and metabolic syndrome participants. Control *n* = 13, metabolic syndrome *n* = 10. Data shown as mean, error bars indicate standard error of the mean. Each symbol represents a participant.

PC:PE ratio has been implicated in cellular and metabolic function in different tissues including skeletal muscle (14, 22). There was no difference in the PC:PE ratio in the SR-enriched muscle fraction between groups (Fig. 6), and there was no significant correlation between PC:PE ratio and any other metabolic measures (supplemental Table S4).

**Fig. 7:**
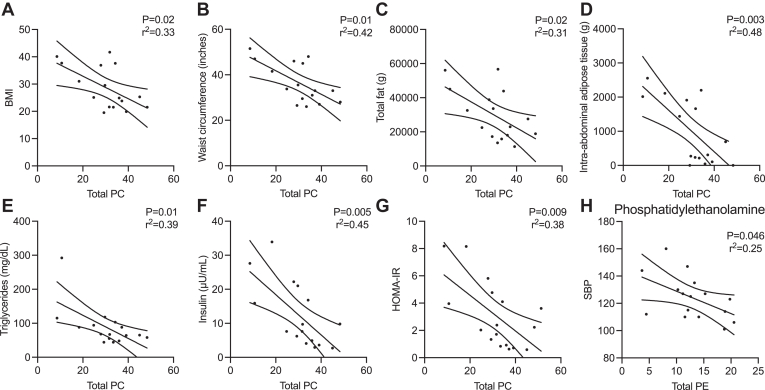
A–G: Correlations between total PC in SR of muscle and BMI, waist circumference, total fat, visceral adipose tissue, serum triglycerides, baseline insulin levels, and HOMA-IR in sedentary controls and sedentary metabolic syndrome subjects (*n* = 16). H: Correlation between total PE in SR of muscle and systolic blood pressure. Each symbol represents a participant, linear regression shown, and dotted lines indicate 95% confidence interval.

Total PC level in the SR-enriched muscle fraction was significantly negatively correlated with BMI, waist circumference, total fat, visceral adipose tissue, triglycerides, baseline insulin, and HOMA-IR (Fig. 7A–G, supplemental Table S4). Total PE level in sarcoplasmic-enriched muscle fraction was significantly negatively correlated with systolic blood pressure (*P* = 0.046, Fig. 7H) but otherwise was not significantly correlated with any other metabolic measure (supplemental Table S4). Total PS level in the SR-enriched muscle fraction did not significantly correlate with any metabolic measures (supplemental Table S4).

## Discussion

Metabolic syndrome is ubiquitous in humans and clearly increases risk for diabetes and its complications (23, 24). The accumulation of lipids is characteristic of metabolic syndrome, and muscle dysfunction is a plausible contributor to diabetes risk, but phospholipids have been neglected as mediators of disease. In liver, PC:PE ratio influenced membrane stability and dysregulation of PC:PE ratio contributed to steatohepatitis (22). Increased PC:PE in liver from obese mice was associated with increased ER stress (25). Skeletal muscle PC:PE has been implicated in insulin sensitivity in humans and mice (14, 16). Previous studies have assessed total muscle phospholipid content, whereas our study specifically assessed SR-enriched fractions to better understand how SR PC:PE might differ between metabolic syndrome and control subjects. We studied sedentary people to avoid possible confounding effects of exercise training on muscle phospholipid content. Because of a prevalence of younger participants in the control group, there was a statistically significant difference in the ages between groups. In model organisms, some phospholipids decrease with age, but major PC species in certain tissues such as brain increase with age (26). In the current study, age was not significantly correlated with total PC, total PE, total PS, or PC to PE ratio. Both total PC and total PE were significantly decreased in muscle SR of participants with metabolic syndrome compared with sedentary controls, but there was no difference in the PC:PE ratio. We observed a significant negative correlation of total PC levels, but not total PE levels or PC:PE ratio, in muscle SR with multiple metabolic parameters suggesting a relationship between SR PC content and insulin resistance.

There were striking and unexpected sex differences in PC and PE with females showing significant decreases and males showing nonsignificant decreases (supplemental Fig. S11). There was no significant difference in clinically determined menopausal status between control and metabolic syndrome subjects, but the sample sizes are small. At least in response to exercise, there are sex differences in muscle fiber type, substrate metabolism, gene expression, and proteomics (27, 28, 29), but data exploring sex differences in phospholipid content are lacking. Estrogen impacts skeletal muscle (30), and women have lower SERCA activity than men (31), but whether skeletal muscle phospholipid composition is involved in these effects is unknown.

Decreased total PC and total PE in muscle SR of participants with metabolic syndrome could be due to altered expression or activity of phospholipase A2 enzymes, which catalyze the hydrolysis of membrane phospholipids at the sn-2 position, releasing free fatty acids and lysoglycerophospholipids. Phospholipase A2 enzymes can be secreted, cytosolic, or membrane bound. These enzymes may be involved in the disruption of insulin signaling, lipid metabolism, mitochondrial function, and GLUT4 translocation in skeletal muscle, all of which contribute to metabolic disease (32). Certain PC and PE species were decreased in skeletal muscle from mice lacking the membrane-bound iPLA2 enzyme group VIB Ca2+-independent phospholipase A2γ, and muscle from these animals showed evidence of mitochondrial dysfunction and increased oxidative stress (33). Phospholipase A2 inhibitors have been shown to improve metabolic end points in animal models (34, 35) and may be important in inflammatory diseases (36).

We isolated phospholipids from the SR, an important mediator of muscle function. Measuring organellar lipid content after subcellular fractionation has yielded insights into the roles of other bioactive lipids in insulin resistance (37, 38, 39). Our study has several limitations, some related to our focus on SR. Because of the mass of muscle tissue required to isolate sufficient SR material, we were unable to assess other end points such as skeletal muscle SERCA. We did not directly measure skeletal muscle insulin sensitivity, but the metabolic syndrome is recognized to be associated with skeletal muscle insulin resistance. Finally, associations of SR phospholipid content in skeletal muscle with other metabolic parameters are correlational and cannot be used to infer causality.

## Conclusion

The observation of decreased SR phospholipid content in skeletal muscle of people with the metabolic syndrome builds on preclinical observations linking SR phospholipid content to muscle insulin sensitivity. Our findings provide a rationale for additional mechanistic investigations to determine if SR phospholipids interact with the regulation of insulin-stimulated glucose disposal in skeletal muscle.

## Data availability

The data supporting this study are available in the main figures, the Supplemental data, or from the corresponding author upon reasonable request.

## Supplemental data

This article contains supplemental data.

## Conflict of interest

The authors declare that they have no conflicts of interest with the contents of this article.
