# Supplementary material for: Decreased sarcoplasmic reticulum phospholipids in human skeletal muscle are associated with metabolic syndrome
Source: J Lipid Res. 2024 Feb 13;65(3):100519. doi: 10.1016/j.jlr.2024.100519 (PMC10937315; doi:10.1016/j.jlr.2024.100519)
Supplement: Supplemental Table S1 [file mmc1.pdf]

SUPPLEMENTAL TABLE 1. Baseline participant characteristics

	Controls n=15	Metabolic Syndrome n=12	P
Age	39.5 ± 16.4	51.3 ± 8.4	0.03
Male	6	5	
Female	9	7	
White	12	8	
Black	3	4	
BMI	23.09 ± 2.70	35.13 ± 3.58	3.41E-10
Waist circumference (inches)	31.25 ± 3.87	45.24 ± 2.98	1.76E-10
SBP	119.4 ± 15.32	135.2 ± 14.2	0.010
DBP	76.9 ± 6.2	84.2 ± 5.2	0.003
Fasting glucose (mg/dL)	85.7 ± 6.9	99.7 ± 11.1	0.0005
Hemoglobin A1c	5.1 ± 0.5	5.4 ± 0.3	0.08
Total cholesterol (mg/dL)	165.7 ± 39.7	186.9 ± 45.6	0.21
Triglycerides (mg/dL)	73.4 ± 23.0	150.0 ± 109.1	0.01
LDL (mg/dL)	94.1 ± 28.6	112.5 ± 30.7	0.12
HDL (mg/dL)	56.8 ± 15.2	47.9 ± 12.4	0.12

Data are presented as mean ± SD.
