# Supplementary material for: Decreased sarcoplasmic reticulum phospholipids in human skeletal muscle are associated with metabolic syndrome
Source: J Lipid Res. 2024 Feb 13;65(3):100519. doi: 10.1016/j.jlr.2024.100519 (PMC10937315; doi:10.1016/j.jlr.2024.100519)
Supplement: Supplemental Table S2 [file mmc2.pdf]

SUPPLEMENTAL TABLE 2. ESI high resolution mass spectra for lipid profiling and structural identification for PC in control samples

m/z	rel. intensity	composition	theo.mass	rdb	[1,2,3]structure	structure.1	structure.2
678.5068	100	C36 H73 O8 N P	678.5068	1.5	PC(28:0)	14:0/14:0-PC	
706.5388	0.32	C38 H77 O8 N P	706.5381	1.5	PC(30:0)	14:0/16:0-PC	
730.539	0.87	C40 H77 O8 N P	730.5381	3.5	PC(32:2)	16:1/16:1-PC	
732.5547	1.44	C40 H79 O8 N P	732.5538	2.5	PC(32:1)	16:0/16:1-PC	
734.5704	1.58	C40 H81 O8 N P	734.5694	1.5	PC(32:0)	16:0/16:0-PC	
742.5754	19.85	C42 H81 O7 N P	742.5745	3.5	PC(p-34:2)	p16:0/18:2-PC	
744.5904	2.42	C42 H83 O7 N P	744.5902	2.5	PC(p-34:1)	p16:0/18:1-PC	a16:0/18:2-PC
746.6063	0.29	C42 H85 O7 N P	746.6058	1.5	PC(a-34:1)	a16:0/18:1-PC	
756.5546	1.76	C42 H79 O8 N P	756.5538	4.5	PC(34:3)	16:1/18:2-PC	16:0/18:3-PC
758.5696	75.99	C42 H81 O8 N P	758.5694	3.5	PC(34:2)	16:0/18:2-PC	
760.5836	12.49	C42 H83 O8 N P	760.5851	2.5	PC(34:1)	16:0/18:1-PC	
766.575	3.5	C44 H81 O7 N P	766.5745	5.5	PC(p-36:4)	p16:0/20:4-PC	
768.5906	3.59	C44 H83 O7 N P	768.5902	4.5	PC(p-36:3)	p18:1/18:2-PC	
770.6063	2.64	C44 H85 O7 N P	770.6058	3.5	PC(p-36:2)	p18:0/18:2-PC	
772.6213	0.32	C44 H87 O7 N P	772.6215	2.5	PC(O-36:2)		
782.5688	7.4	C44 H81 O8 N P	782.5694	5.5	PC(36:4)	16:0/20:4-PC	18:2/18:2-PC
784.5853	10.72	C44 H83 O8 N P	784.5851	4.5	PC(36:3)	18:1/18:2-PC	16:0/20:3-PC
786.6007	18.8	C44 H85 O8 N P	786.6007	3.5	PC(36:2)	18:0/18:2-PC	18:1/18:1-PC
788.609	2.27	C44 H87 O8 N P	788.6164	2.5	PC(36:1)	18:0/18:1-PC	
792.5902	1.35	C46 H83 O7 N P	792.5902	6.5	PC(O-38:6)		
794.606	0.96	C46 H85 O7 N P	794.6058	5.5	PC(p-38:4)	p18:0/20:4-PC	
796.6211	0.19	C46 H87 O7 N P	796.6215	4.5	PC(a-38:4)	a18:0/20:4-PC	
806.5705	1.77	C46 H81 O8 N P	806.5694	7.5	PC(38:6)	16:0/22:6-PC	18:2/20:4-PC
808.5862	3.42	C46 H83 O8 N P	808.5851	6.5	PC(38:5)	18:1/20:4-PC	16:0/22:5-PC
810.6009	2.23	C46 H85 O8 N P	810.6007	5.5	PC(38:4)	18:0/20:4-PC	
812.6161	0.37	C46 H87 O8 N P	812.6164	4.5	PC(38:3)	18:0/20:3-PC	18:1/20:2-PC
834.6008	0.19	C48 H85 O8 N P	834.6007	7.5	PC(40:6)	18:0/22:6-PC	18:2/22:4-PC
836.6164	0.13	C48 H87 O8 N P	836.6164	6.5	PC(40:5)	18:0/22:5-PC	

[1] Structures are extracted from home-built lipid database established by CID tandem mass spectrometry.
[2] In the main text, only major PC species (and only those assigned to structures) are subjected for quantitation.
[3] Abbreviations "p" is plasmalogen: 1-O-alkenyl-, "a" is plasmanyl: 1-O-alkyl-, "O" is plasmanyl/plasmenyl not defined.
