# Supplementary material for: Decreased sarcoplasmic reticulum phospholipids in human skeletal muscle are associated with metabolic syndrome
Source: J Lipid Res. 2024 Feb 13;65(3):100519. doi: 10.1016/j.jlr.2024.100519 (PMC10937315; doi:10.1016/j.jlr.2024.100519)
Supplement: Supplemental Table S3 [file mmc3.pdf]

SUPPLEMENTAL TABLE 3. ESI high resolution mass spectra for lipid profiling and structural identification for PE in control samples

m/z	rel. intensity	composition	theo. mass	rdb	[1,2,3]structure	[1]structure.1	structure.2
634.4453	100.00	C33 H65 O8 N P	634.4453	2.5	PE(28:0)	14:0/14:0-PE	
698.513	13.03	C39 H73 O7 N P	698.513	4.5	PE(P-34:2)	p16:0/18:2-PE	
700.5287	11.35	C39 H75 O7 N P	700.5287	3.5	PE(P-34:1)	p16:0/18:1-PE	
714.5079	8.99	C39 H73 O8 N P	714.5079	4.5	PE(34:2)	16:0/18:2-PE	16:1/18:1-PE
716.5236	15.96	C39 H75 O8 N P	716.5236	3.5	PE(34:1)	16:0/18:1-PE	
722.5127	32.34	C41 H73 O7 N P	722.513	6.5	PE(P-36:4)	p16:0/20:4-PE	
724.5285	7.14	C41 H75 O7 N P	724.5287	5.5	PE(P-36:3)	p16:0/20:3-PE	
726.5441	13.42	C41 H77 O7 N P	726.5443	4.5	PE(P-36:2)	p18:0/18:2-PE	p18:1/18:1-PE
738.5078	9.51	C41 H73 O8 N P	738.5079	6.5	PE(36:4)	16:0/20:4-PE	18:1/18:3-PE
740.5236	6.97	C41 H75 O8 N P	740.5236	5.5	PE(36:3)	18:1/18:2-PE	16:0/20:3-PE
742.5392	27.44	C41 H77 O8 N P	742.5392	4.5	PE(36:2)	18:0/18:2-PE	
744.555	16.65	C41 H79 O8 N P	744.5549	3.5	PE(36:1)	18:0/18:1-PE	
748.5287	19.10	C43 H75 O7 N P	748.5287	7.5	PE(P-38:5)	p16:0/22:5-PE	
750.5443	33.94	C43 H77 O7 N P	750.5443	6.5	PE(P-38:4)	p18:0/20:4-PE	
764.5238	7.18	C43 H75 O8 N P	764.5236	7.5	PE(38:5)	18:1/20:4-PE	16:0/22:5-PE
766.5392	26.06	C43 H77 O8 N P	766.5392	6.5	PE(38:4)	18:0/20:4-PE	16:0/22:4-PE
770.5705	6.62	C43 H81 O8 N P	770.5705	4.5	PE(38:2)	18:0/20:2-PE	
778.575	5.20	C45 H81 O7 N P	778.5756	6.5	PE(P-40:4)	p18:0/22:4-PE	
792.555	5.76	C45 H79 O8 N P	792.5549	7.5	PE(40:5)	18:0/22:5-PE	18:1/22:4-PE
794.5705	7.66	C45 H81 O8 N P	794.5705	6.5	PE(40:4)	18:0/22:4-PE	

[1] Structures are extracted from home-built lipid database established by CID tandem mass spectrometry.
[2] In the main text, only major PE species (and only those assigned structures) are subjected for quantitation.
[3] Abbreviation “p” is plasmalogen: 1-O-alkenyl-.
