# Supplementary material for: Decreased sarcoplasmic reticulum phospholipids in human skeletal muscle are associated with metabolic syndrome
Source: J Lipid Res. 2024 Feb 13;65(3):100519. doi: 10.1016/j.jlr.2024.100519 (PMC10937315; doi:10.1016/j.jlr.2024.100519)
Supplement: Supplemental Table S4 [file mmc4.pdf]

SUPPLEMENTAL TABLE 4. Correlations of lipid species with metabolic variables

	Total PC			Total PE			PC:PE	Total PS
	P	R^2	Slope	P	R^2	Slope	P	P
Age	0.66			0.90			0.62	0.18
BMI	**0.02**	0.33	-0.41	0.11			0.57	0.20
Waist circumference (inches)	**0.01**	0.42	-0.52	0.08	0.20	-0.78	0.48	0.17
SBP	0.10			**0.046**	0.25	-1.64	0.66	0.44
DBP	0.10			0.11			0.88	0.56
Fasting glucose (mg/dL)	0.06	0.22	-0.51	0.32			0.26	0.29
Hemoglobin A1c	0.19			0.33			0.66	0.22
Total cholesterol (mg/dL)	0.93			0.39			0.20	0.66
LDL (mg/dL)	0.99			0.31			0.13	0.98
HDL (mg/dL)	0.16			0.43			0.49	0.12
Triglycerides (mg/dL)	**0.01**	0.39	-3.41	0.07	0.21	-5.47	0.93	0.53
Percent fat, %, Total body	0.11			0.33			0.38	0.58
Percent fat, %, Legs	0.82			0.74			0.93	0.51
Percent fat, %, Trunk	**0.03**	0.28	-0.59	0.26			0.24	0.81
Percent fat, %, android	**0.03**	0.31	-0.78	0.25			0.24	0.96
Percent fat, %, gynoid	0.47			0.67			0.50	0.58
Android/gynoid fat ratio	**0.01**	0.35	-0.02	0.24			0.29	0.66
Total fat (g)	**0.02**	0.31	-762.70	0.12			0.56	0.20

Total lean (g)	0.09	0.19	-488.90	0.23			0.91	0.12
Bone mineral content (g)	0.47			0.73			0.99	0.21
Fat mass ratio, Trunk/total	**0.03**	0.30	-0.01	0.34			0.18	0.88
Fat mass ratio, Legs/total	0.06	0.23	0.00	0.40			0.28	0.68
Fat mass ratio, Limbs/trunk	0.08	0.20	0.02	0.40			0.36	0.64
VAT (g)	**0.003**	0.48	-60.76	0.11			0.29	0.61
AUC insulin	0.14			0.22			0.68	0.41
AUC OGTT	0.87			0.82			0.77	0.19
Baseline insulin (µU/mL)	**0.005**	0.45	-0.59	0.05	0.24	-0.95	0.35	0.23
