# Supplementary material for: Decreased sarcoplasmic reticulum phospholipids in human skeletal muscle are associated with metabolic syndrome
Source: J Lipid Res. 2024 Feb 13;65(3):100519. doi: 10.1016/j.jlr.2024.100519 (PMC10937315; doi:10.1016/j.jlr.2024.100519)
Supplement: Supplemental Figure S1 [file mmc5.pdf]

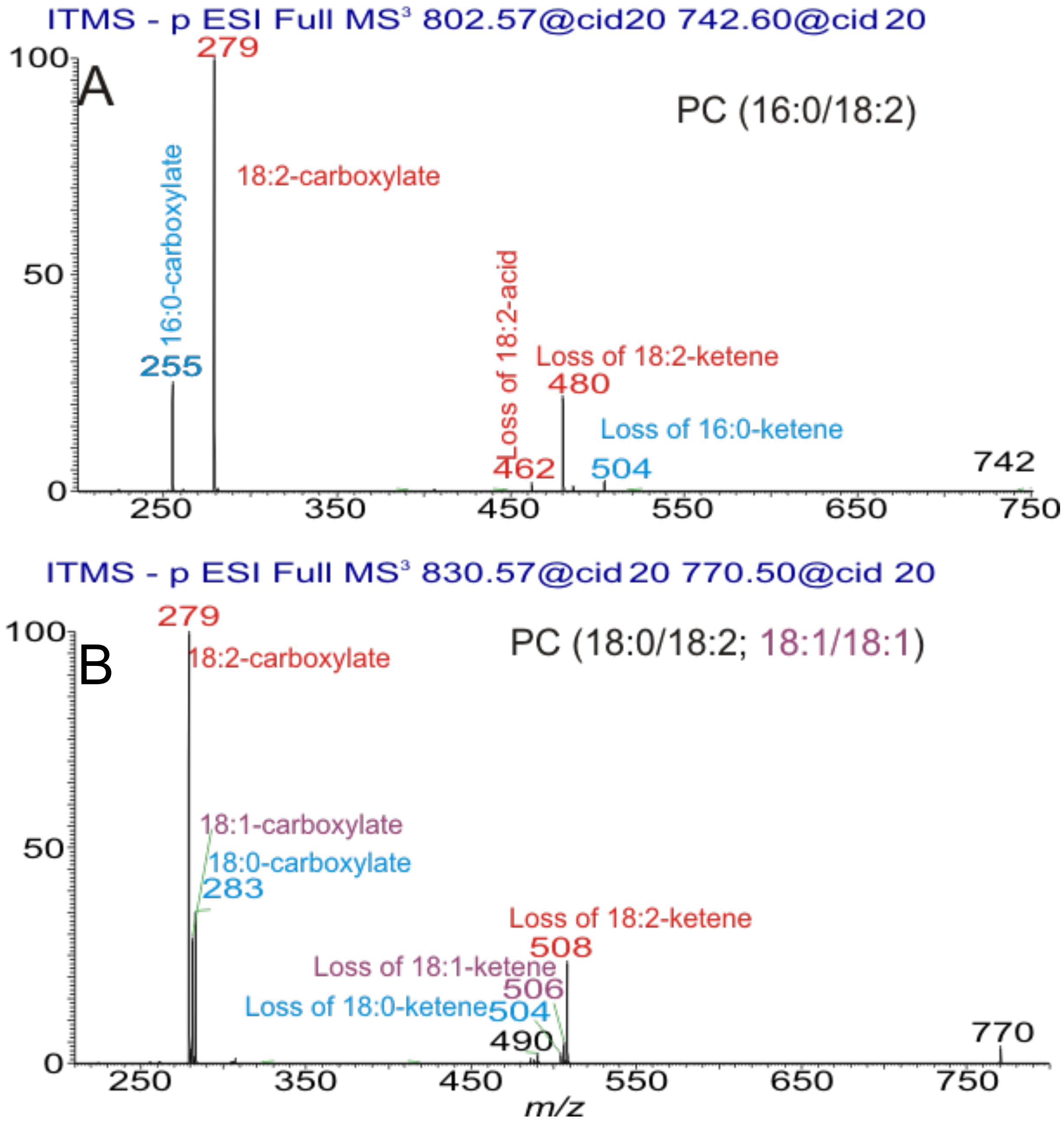

Fig. S1. (A) LIT MS[3] spectrum of the ion at m/z 742 (802 →742) from the $[M+ HCO_2]^-$ ion of PC (16:0/18:2) that first renders loss of HCO_2CH_3 to become N-dimethyl PE, which further dissociates to yield a PE-like MS[3] spectrum that readily locates the identity and regiospecificity of the fatty acyl chains. (B) is the analogous spectrum arising from the $[M+ HCO_2]^-$ ion of PC (18:0/18:2) at m/z 830 that renders same HCO_2CH_3 loss to m/z 770, which undergoes further dissociation to yield a similar PE-like spectrum and provide structural information leading to assign a major PC (18:0/18:2) structure. In addition, an ion at m/z 281, representing a 18:1-carboxylate anion is also present, consistent with the ion of m/z 506 from loss of 18:1-ketene, indicating the presence of a minor 18:1/18:1-PC isomer.
