# Supplementary material for: Decreased sarcoplasmic reticulum phospholipids in human skeletal muscle are associated with metabolic syndrome
Source: J Lipid Res. 2024 Feb 13;65(3):100519. doi: 10.1016/j.jlr.2024.100519 (PMC10937315; doi:10.1016/j.jlr.2024.100519)
Supplement: Supplemental Figure S2 [file mmc6.pdf]

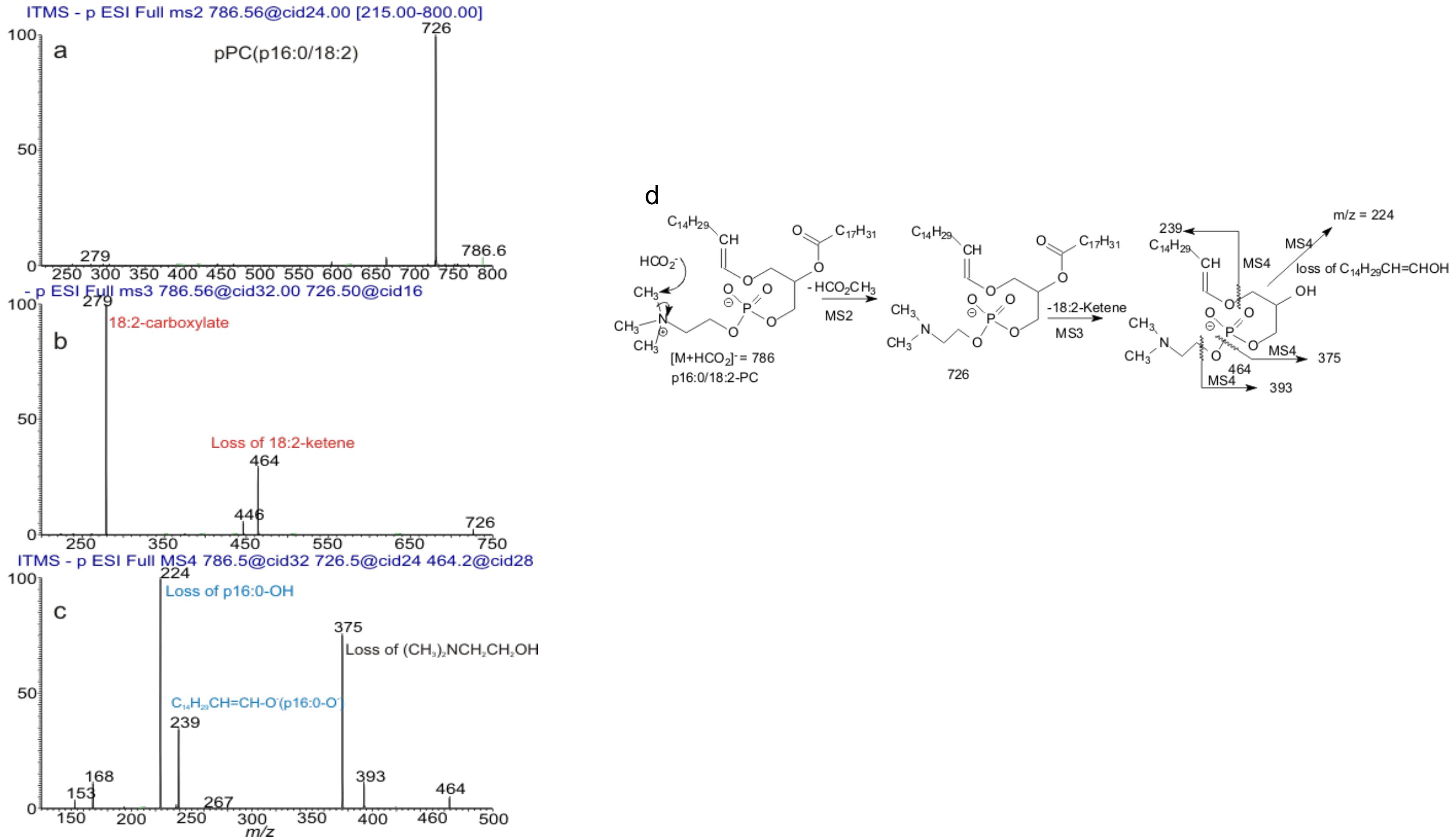

Fig. S2. The linear ion trap MS[2] spectrum of the $[M+ HCO_2]^-$ ion of p16:0/18:2-PC at m/z 786 (A), its MS[3] spectrum of m/z 726 (786 →726) (B), and MS[4] spectrum of m/z 464 (786 →726 →464) (C) defining the p16:0/18:2-PC structure. (D) Schematic of the fragmentation processes leading to the structure identification. The $[M+ CH_3CO_2]^-$ adduct ion of p16:0/18:2-PC at m/z 786 undergoes prompt dissociation to m/z 726 by loss of $CH_3CO_2CH_3$ (A). The ion of m/z 726 is equivalent to a 1-O-alkenyl-2-acyl phospho-N,N-dimethylethanolamine, which further dissociates to m/z 464 by loss of 18:2-fatty acid substituent at sn-2 as a ketene (B). The MS[4] spectrum (786→726→464, C) contains feature fragment ions that are previously reported for plasmalogen PC (Hsu et al. 2014), defining a p16:0/18:2-PC structure.
