# Supplementary material for: Decreased sarcoplasmic reticulum phospholipids in human skeletal muscle are associated with metabolic syndrome
Source: J Lipid Res. 2024 Feb 13;65(3):100519. doi: 10.1016/j.jlr.2024.100519 (PMC10937315; doi:10.1016/j.jlr.2024.100519)
Supplement: Supplemental Figure S3 [file mmc7.pdf]

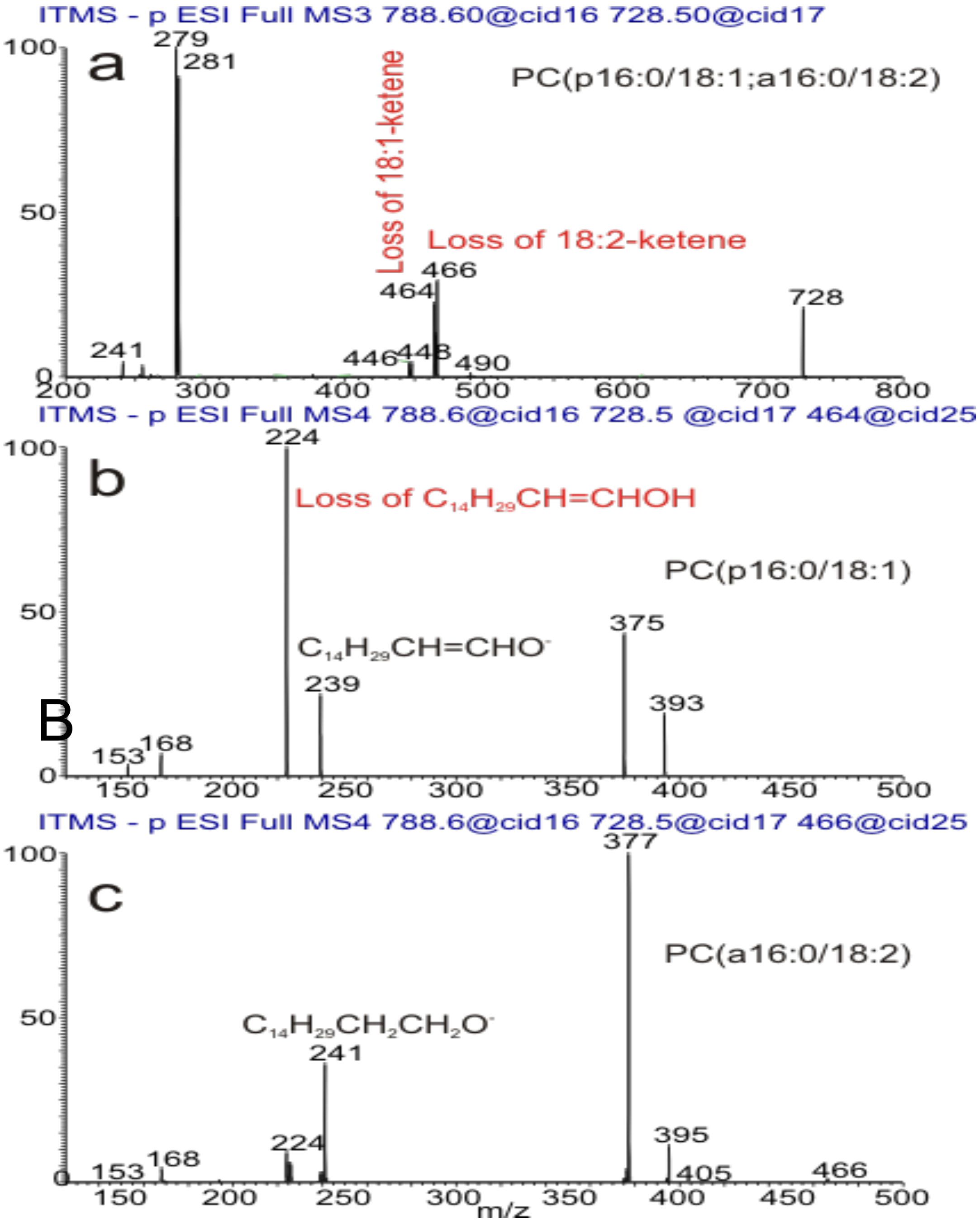

Fig. S3. The $[M+ HCO_2]^-$ ion of p16:0/18:2-PC at m/z 788 (not shown) was subjected to MS^3 spectrum of m/z 728 (788 →728) (A), and MS^4 spectrum of m/z 464 (788 →728 →464) (B) that defined a p16:0/18:1-PC structure. In Panel A, the ion at m/z 466 from loss of 18:1-ketene is also present. The MS^4 spectrum of m/z 466 (788 →728 →466) (C) showed a spectrum pattern that is typical of a plasmanyl PC, leading to define an a16:0/18:2-PC structure. This exemplifies the presence of a plasmanyl/plasmenyl PC isomer for the same ion.
