# Supplementary material for: Decreased sarcoplasmic reticulum phospholipids in human skeletal muscle are associated with metabolic syndrome
Source: J Lipid Res. 2024 Feb 13;65(3):100519. doi: 10.1016/j.jlr.2024.100519 (PMC10937315; doi:10.1016/j.jlr.2024.100519)
Supplement: Supplemental Figure S4 [file mmc8.pdf]

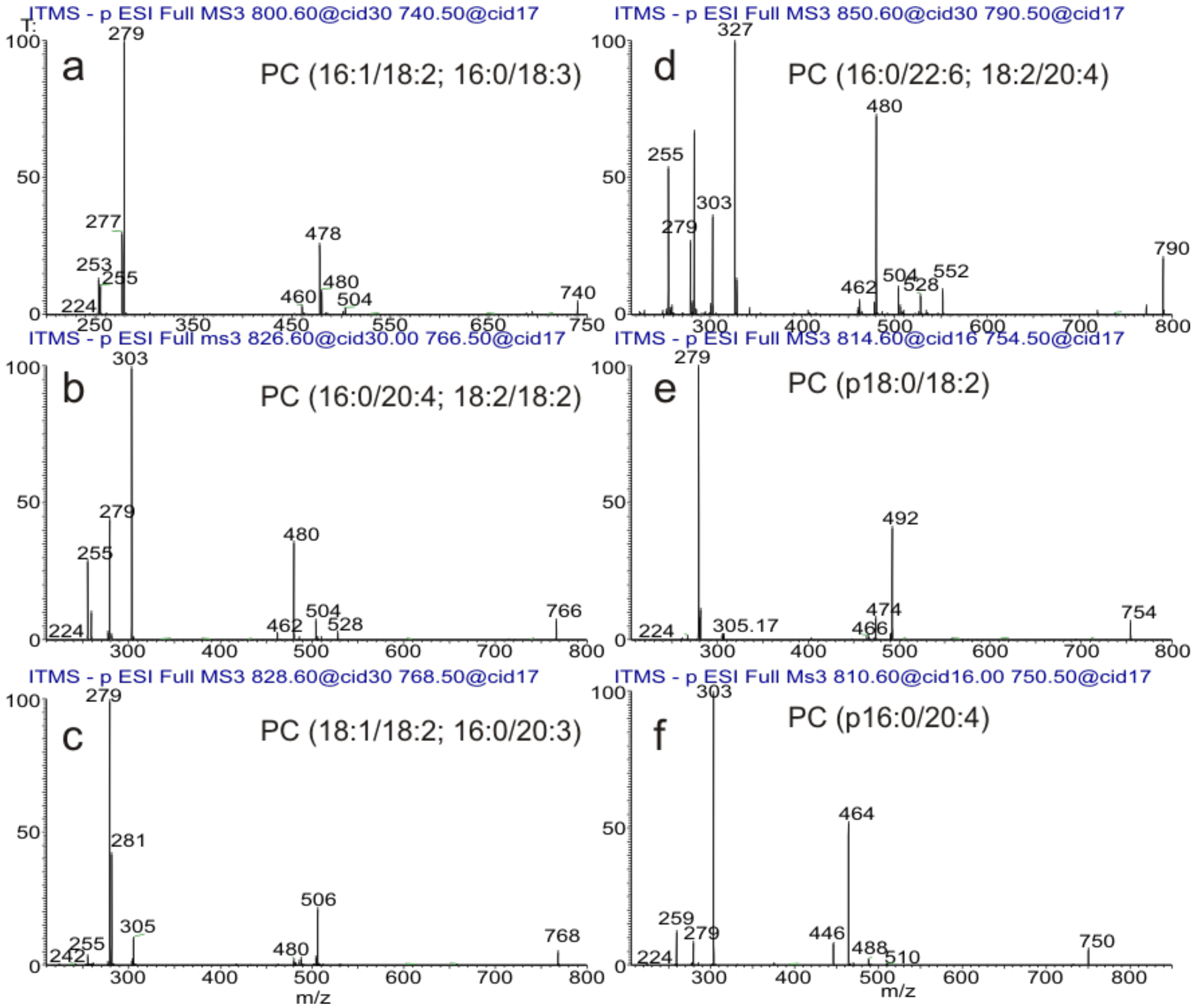

Fig. S4. The LIT MS^3 spectra of (A) m/z 740 (800 → 740) from the $[M+HCO_2]^-$ ion of m/z 800 (equivalent to $[M+H]^+$ at m/z 756), (B) m/z 766 (826 → 766) (equivalent to $[M+H]^+$ at m/z 782), (C) m/z 768 (828 → 768) (equivalent to $[M+H]^+$ at m/z 784), (D) m/z 790 (850 → 790) (equivalent to $[M+H]^+$ at m/z 806), (E) m/z 754 (814 → 754) (equivalent to $[M+H]^+$ at m/z 770), and (F) m/z 750 (810 → 750) (equivalent to $[M+H]^+$ at m/z 756). These spectra were obtained from the major PC species observed in the lipid extract, and represent (16:1/18:2-PC; 16:0/18:3-PC) for A, (16:0/20:4-PC; 18:2/18:2-PC) for B, (18:1/18:2-PC; 16:0/20:3-PC) for C, (16:0/22:6-PC; 18:2/20:4-PC) for D, p18:0/18:2-PC for E, and p16:0/20:4-PC for F. The 1-o-alkyl-, 1-O-alkenyl-, and 1-O acyl chains at sn-1 were confirmed by MS^4 spectrum as indicated by the diagram in Fig. S2D.
