# Supplementary material for: Decreased sarcoplasmic reticulum phospholipids in human skeletal muscle are associated with metabolic syndrome
Source: J Lipid Res. 2024 Feb 13;65(3):100519. doi: 10.1016/j.jlr.2024.100519 (PMC10937315; doi:10.1016/j.jlr.2024.100519)
Supplement: Supplemental Figure S5 [file mmc9.pdf]

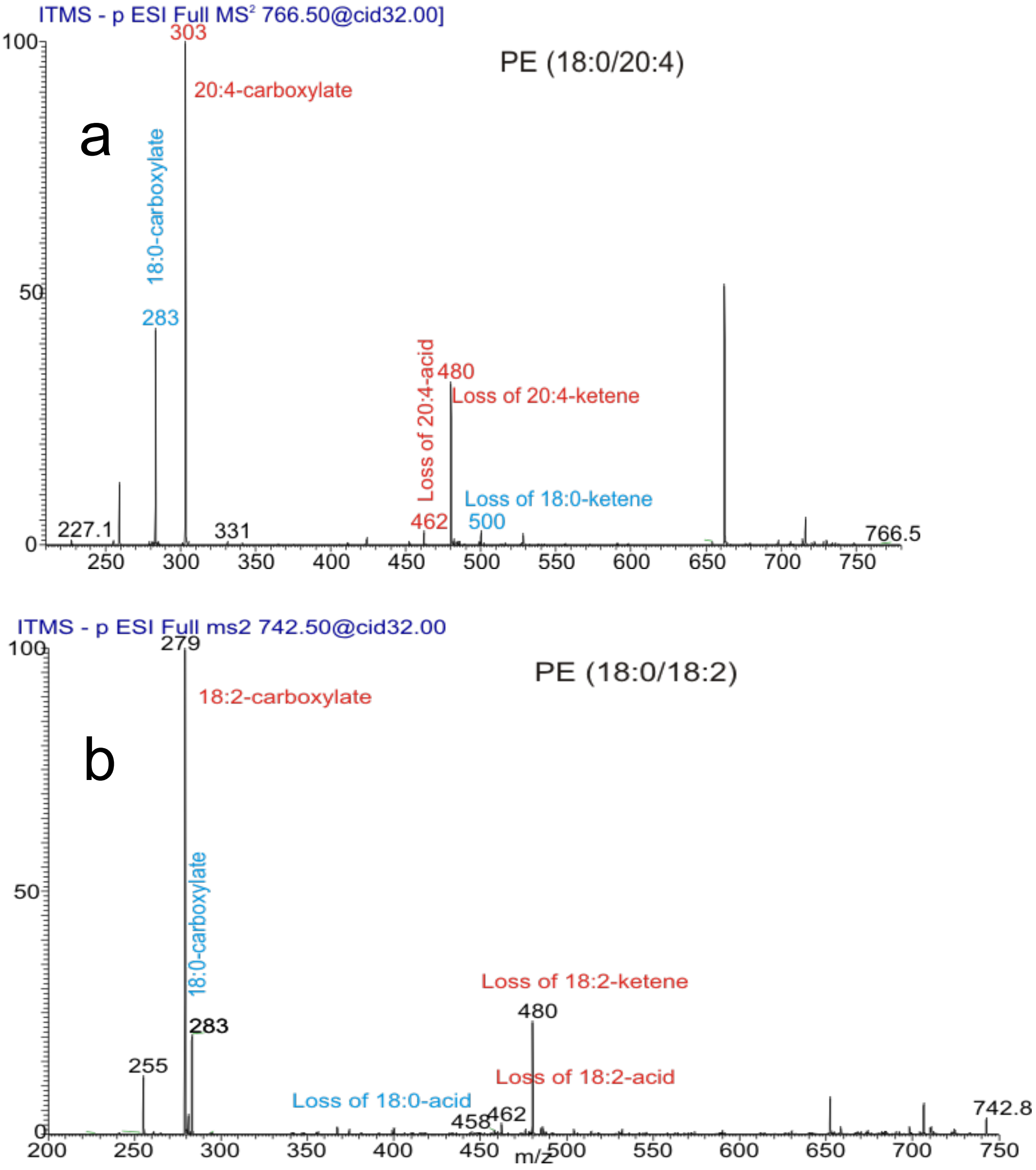

Fig. S5. (A) LIT MS^2 spectrum of $[M-H]^-$ ion at m/z 766 that led to assign PE (18:0/20:4) structure. Ions at m/z 480 and 462 arise from loss of 20:4 FA at sn2, together with an abundant ion at m/z 303 representing 20:4-FA anion. The ions at m/z 500 and 482 arise from loss of 18:0-FA substituent at sn-1, consistent with the presence of 18:0-FA anion at m/z 283. The regiospecificity is established by the abundances of the fragment ions (i.e., ions from loss of FA at sn-2 are more abundant). (B) LIT MS^2 spectrum of $[M-H]^-$ ion at m/z 742 that led to assign PE (18:0/18:2) structure. Ions at m/z 480 and 462 arise from loss of 18:2 FA as ketene and acid at sn2, respectively, and the abundant ion at m/z 279 represents an 18:2-FA anion. The ions at m/z 476 and 458 arise from analogous loss of 18:0-FA substituent at sn-1, and the ion at 283 represents an 18:0-FA anion. The regiospecificity is established by the fact that the ion at m/z 480 from loss of 18:2-FA at sn-2 is more abundant than m/z 476 (very low abundance; not shown).
