# Supplementary material for: Decreased sarcoplasmic reticulum phospholipids in human skeletal muscle are associated with metabolic syndrome
Source: J Lipid Res. 2024 Feb 13;65(3):100519. doi: 10.1016/j.jlr.2024.100519 (PMC10937315; doi:10.1016/j.jlr.2024.100519)
Supplement: Supplemental Figure S6 [file mmc10.pdf]

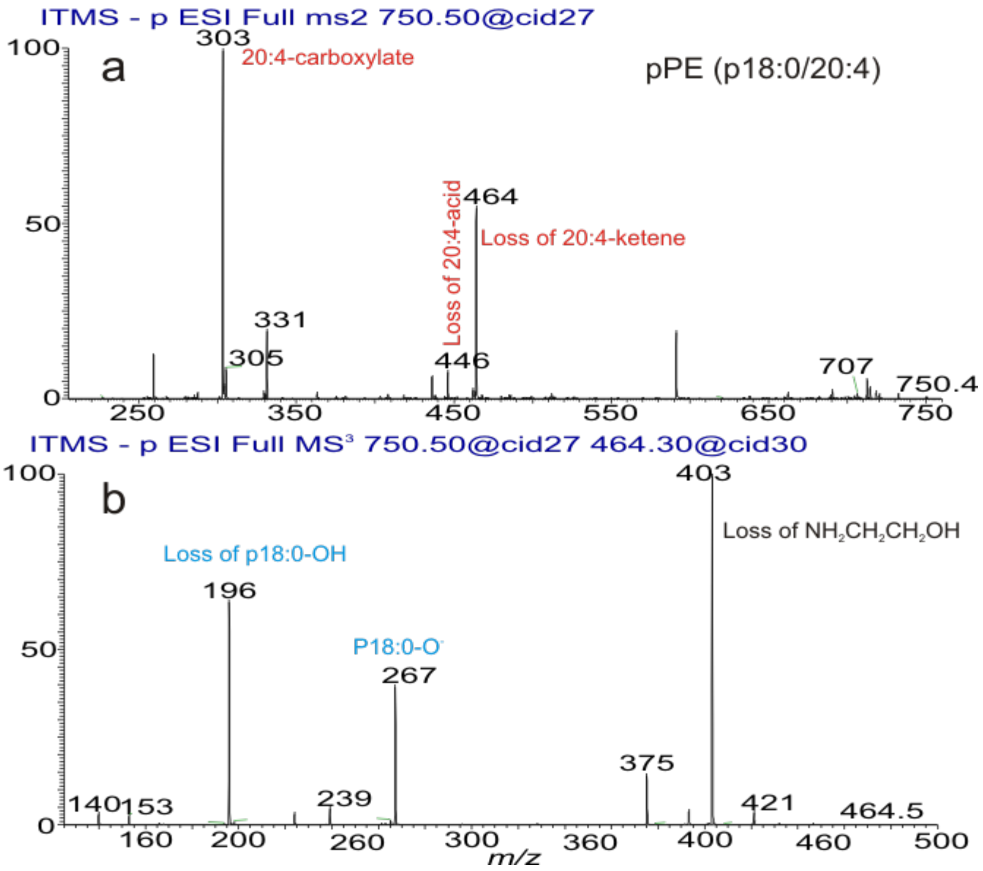

Fig. S6. (A) The LIT MS^2 spectrum of $[M-H]^-$ ion at m/z 750 that led to assign the pPE (p18:0/20:4) structure. Ions at m/z 464 and 446 arise from loss of 20:4 FA at sn2 and the abundant ion at m/z 303 represents a 20:4-FA anion. (B) To verify the presence of an alkenyl residue at sn-1, a MS^3 spectrum of m/z 464 (750→464) was obtained, and the spectrum contained an alkeoxide ion at m/z 267 along with ions at m/z 403 (loss of ethanolamine), and ions at m/z 196 from loss of the alkenol residue, a diagnostic ion for a plasmalogen PE.
