# Supplementary material for: Decreased sarcoplasmic reticulum phospholipids in human skeletal muscle are associated with metabolic syndrome
Source: J Lipid Res. 2024 Feb 13;65(3):100519. doi: 10.1016/j.jlr.2024.100519 (PMC10937315; doi:10.1016/j.jlr.2024.100519)
Supplement: Supplemental Figure S7 [file mmc11.pdf]

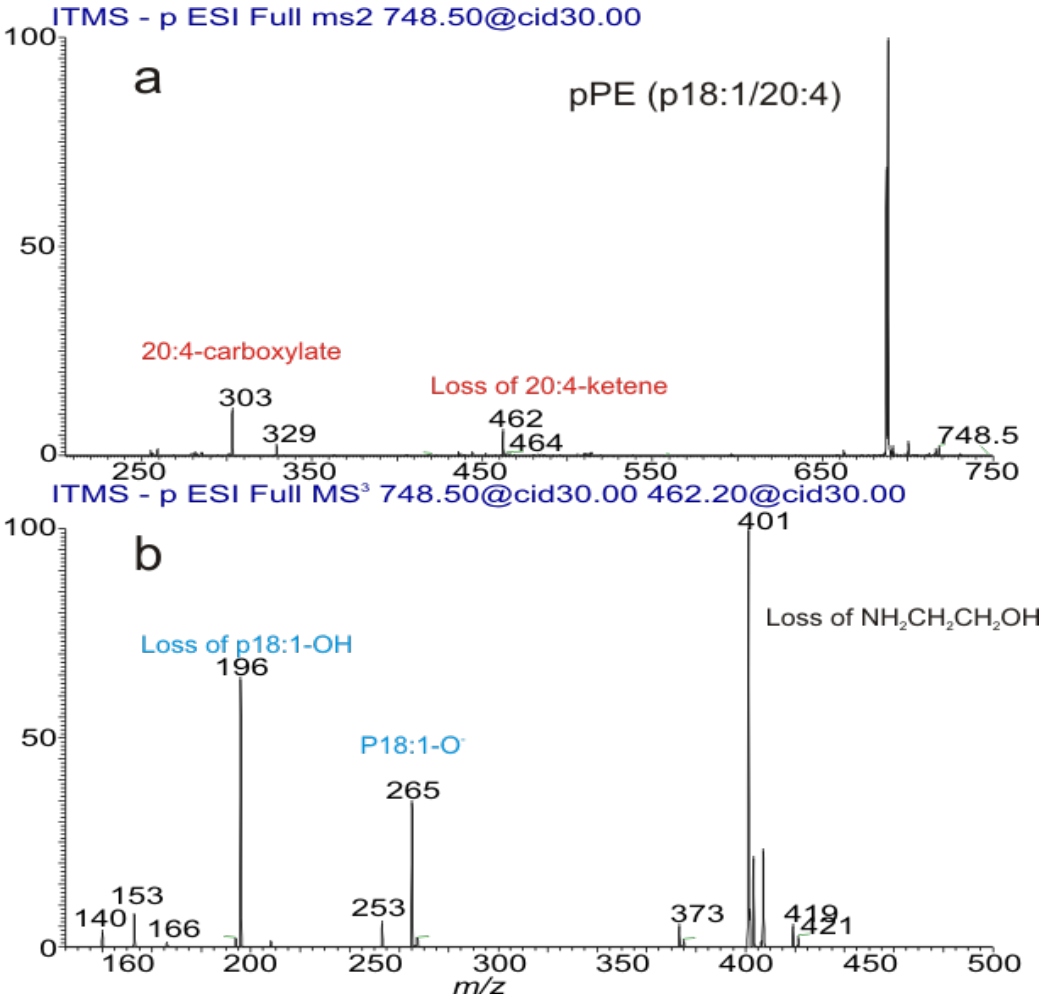

Fig. S7. (A) The LIT MS^2 spectrum of the $[M – H]^-$ ion at m/z 748 that led to assign the pPE (p18:1/20:4) structure. Ions at m/z 462 arise from loss of 20:4 FA at sn2 and the ion at m/z 303 represents a 20:4-FA anion. (B) To verify the presence of the alkenyl residue at sn-1, a MS^3 spectrum of m/z 462 (748→462) was obtained, and the spectrum contained an alkeoxide anion ($R_1CH=CH-O^-$) at m/z 265 along with ions at m/z 401 (loss of ethanolamine) and m/z 196 arising from loss of $R_1CH=CH-OH$ residue (alkenol), a diagnostic ion for a plasmalogen PE.
