# Supplementary material for: Decreased sarcoplasmic reticulum phospholipids in human skeletal muscle are associated with metabolic syndrome
Source: J Lipid Res. 2024 Feb 13;65(3):100519. doi: 10.1016/j.jlr.2024.100519 (PMC10937315; doi:10.1016/j.jlr.2024.100519)
Supplement: Supplemental Figure S8 [file mmc12.pdf]

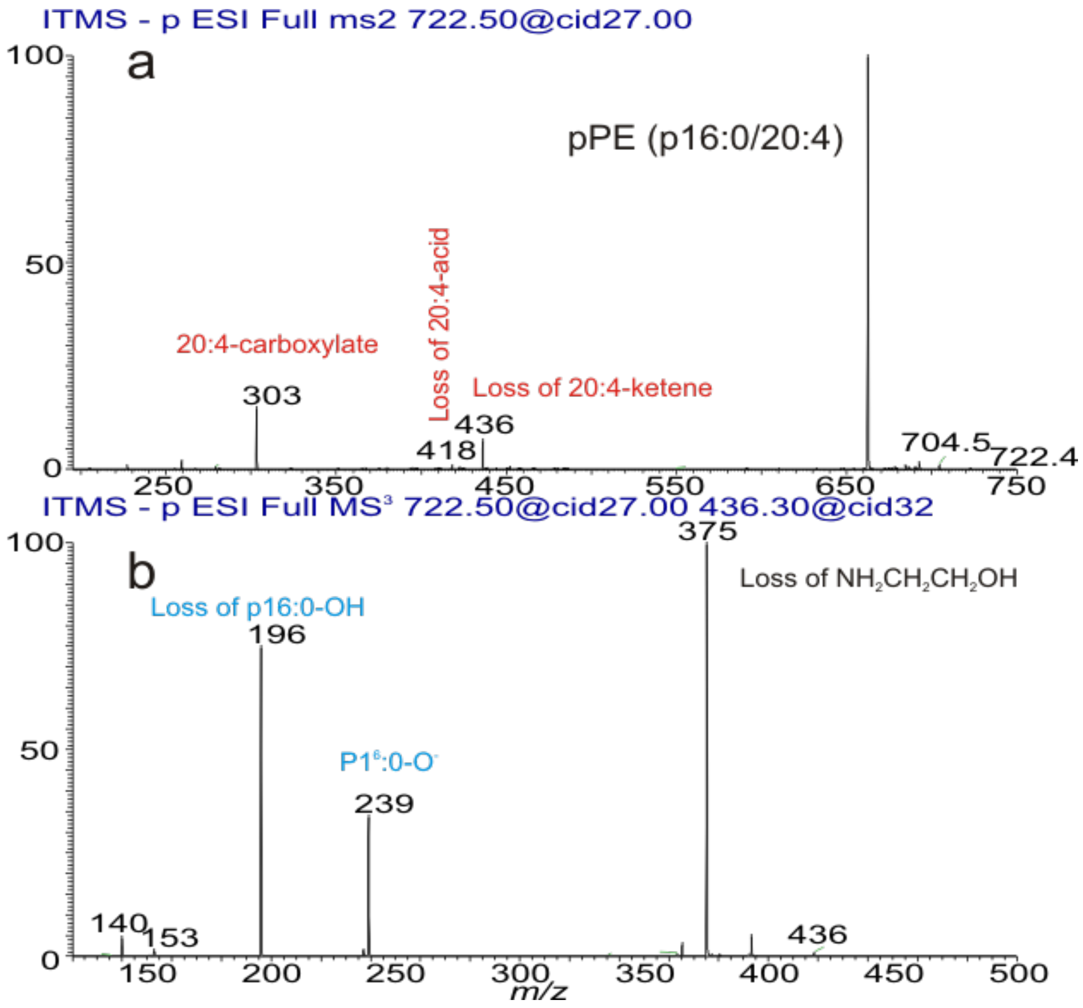

Fig. S8. (A) The LIT MS^2 spectrum of [M – H]$^-$ ion at m/z 722 that led to assign the pPE (p16:0/20:4) structure. Ions at m/z 436 arise from loss of 20:4 FA at sn-2 and the ion at m/z 303 represents a 20:4-FA anion. (B) To verify the presence of an 1-O-alkenyl (R_1CH=CH-O) residue at sn-1, a MS^3 spectrum of m/z 436 (722→436) was obtained, and the spectrum contained an R_1CH=CH-O$^-$ ion at m/z 239 along with ions at m/z 475 (loss of ethanolamine), and ions at m/z 196 arising from loss of R_1CH=CH-OH residue (alkenol), a diagnostic ion for a plasmalogen PE.
