# Supplementary material for: Decreased sarcoplasmic reticulum phospholipids in human skeletal muscle are associated with metabolic syndrome
Source: J Lipid Res. 2024 Feb 13;65(3):100519. doi: 10.1016/j.jlr.2024.100519 (PMC10937315; doi:10.1016/j.jlr.2024.100519)
Supplement: Supplemental Figure S9 [file mmc13.pdf]

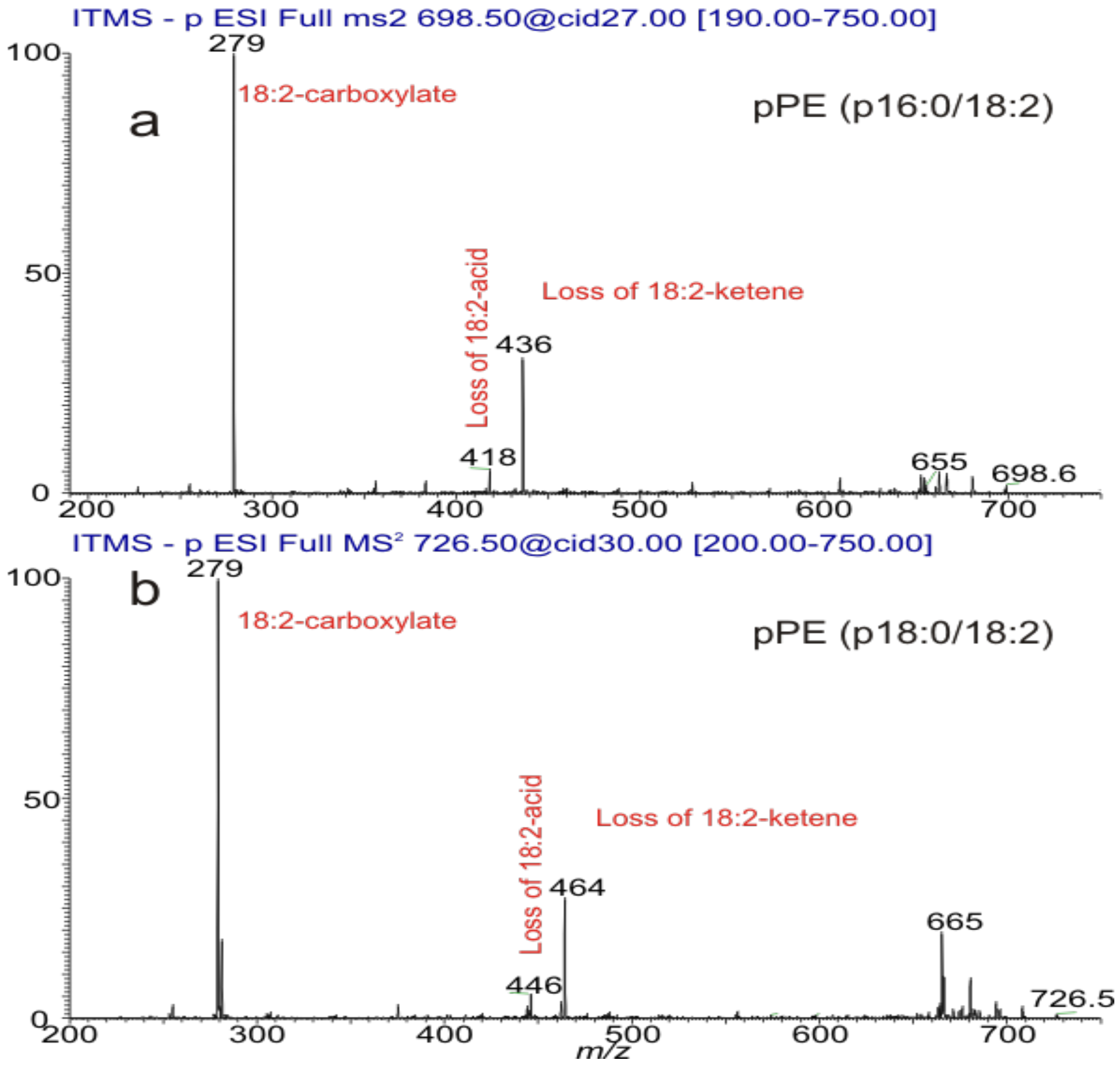

Fig. S9. (A) The LIT MS^2 spectrum of $[M – H]^-$ ion at m/z 698 that led to assign the pPE (p16:0/18:2) structure. Ions at m/z 436 arise from loss of 18:2 FA at sn2 and the ion at m/z 279 represents a 18:2-FA anion. To verify the presence of alkenyl residue at sn-1, a MS^3 spectrum of m/z 436 (698→436) was identical to Fig. S8B indicating the presence of an identical p16:0 at sn-1. (B) The LIT MS^2 spectrum of $[M – H]^-$ ion at m/z 726 that led to assign the pPE (p18:0/18:2) structure. Ions at m/z 464 arise from loss of 18:2 FA at sn-2 and the ion at m/z 279 represents a 18:2-FA anion. The MS^3 spectrum of m/z 464 (726→464) was identical to Fig. S6B indicating the presence of an identical p18:0 chain at sn-1.
