# Supplementary material for: Decreased sarcoplasmic reticulum phospholipids in human skeletal muscle are associated with metabolic syndrome
Source: J Lipid Res. 2024 Feb 13;65(3):100519. doi: 10.1016/j.jlr.2024.100519 (PMC10937315; doi:10.1016/j.jlr.2024.100519)
Supplement: Supplemental Figure S10 [file mmc14.pdf]

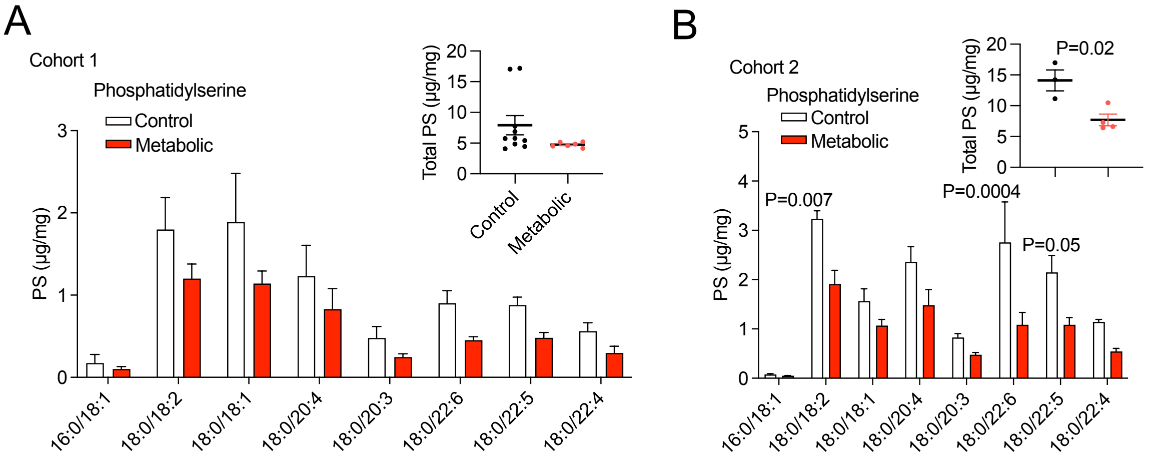

Fig. S10. Phosphatidylserine (PS) levels in sarcoplasmic reticulum of muscle in Cohort 1 (A) control n=10, metabolic n=6, and Cohort 2 (B) control n=3, metabolic n=4. Data shown as mean, error bars indicate standard error of the mean. 2 way ANOVA with Bonferroni multiple comparison test. Insets show total PS levels, data shown as mean, error bars represent standard error of the mean, Mann-Whitney test, each symbol representing a participant sample.
