# Supplementary material for: Decreased sarcoplasmic reticulum phospholipids in human skeletal muscle are associated with metabolic syndrome
Source: J Lipid Res. 2024 Feb 13;65(3):100519. doi: 10.1016/j.jlr.2024.100519 (PMC10937315; doi:10.1016/j.jlr.2024.100519)
Supplement: Supplemental Figure S11 [file mmc15.pdf]

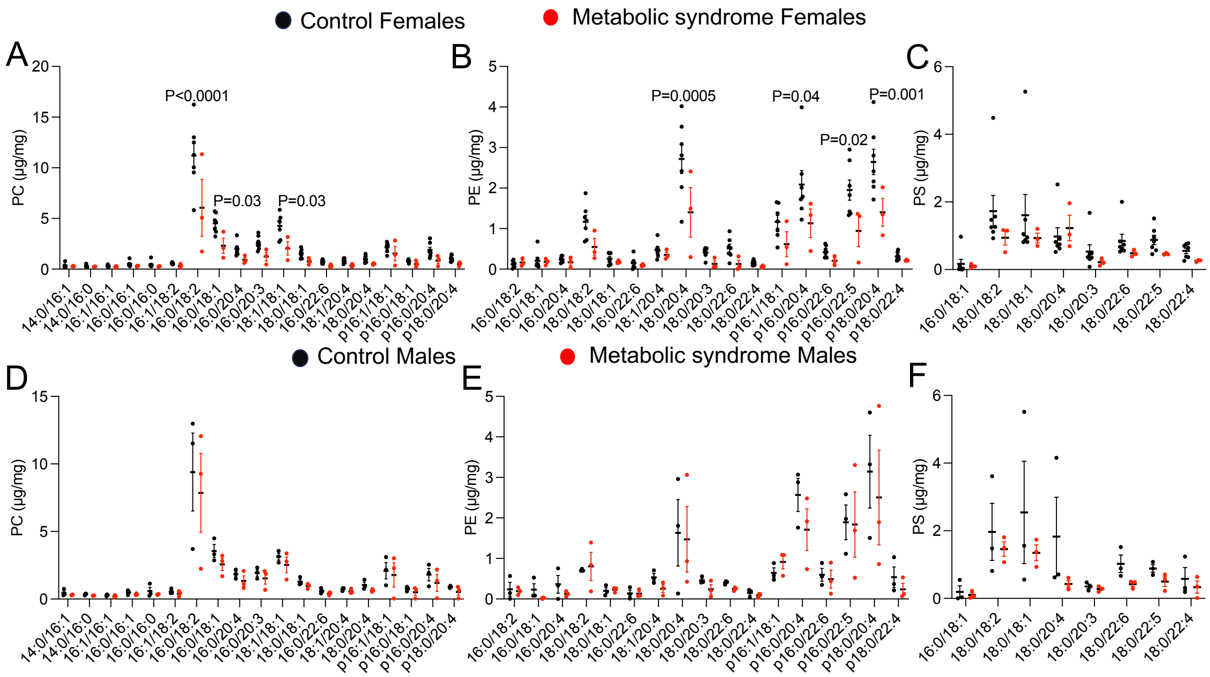

Fig. S11. Phosphatidylcholine (PC) levels (A,D), phosphatidylethanolamine (PE) levels (B,E), and phosphatidylserine (PS) levels (C,F) from female (A-C) and male (D-F) participants in Cohort 1. Data shown as mean, error bars represent standard error of the mean, each symbol represents a participant sample. Data analyzed by 2 way ANOVA with Bonferroni multiple comparison test.
